# Supplementary material for: Conformational Properties of Flaxseed Rhamnogalacturonan-I and Correlation between Primary Structure and Conformation
Source: Polymers (Basel). 2022 Jun 30;14(13):2667. doi: 10.3390/polym14132667 (PMC9269093; doi:10.3390/polym14132667)
Supplement: Supplementary file 1 [file polymers-14-02667-s001.zip › polymers-1750001-supplementary.pdf]

Supplementary Material

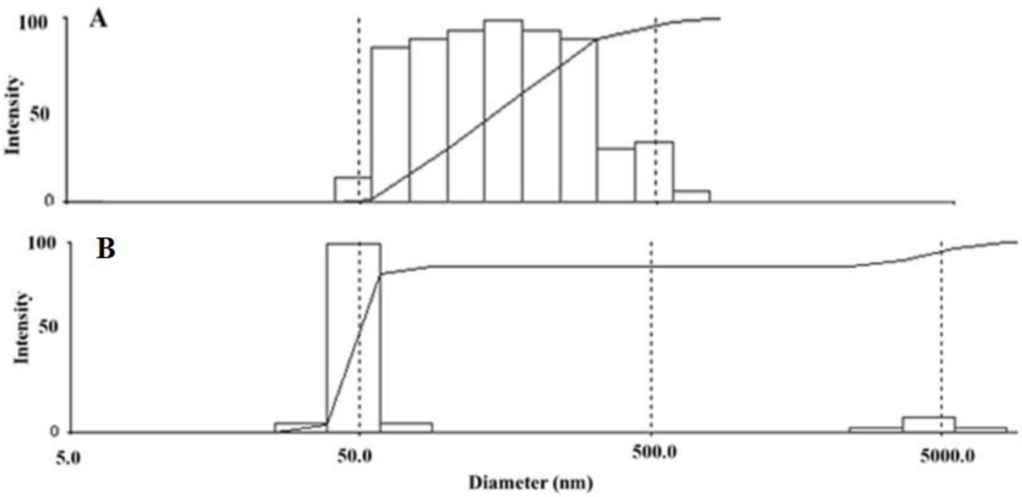

Figure S1. Molecular size distribution of flaxseed kernel RG-I: (A) Milli-Q water, and (B) 0.5 M KOH determined by dynamic light scattering (concentration: 0.1 mg/mL, temperature: 23 ºC).

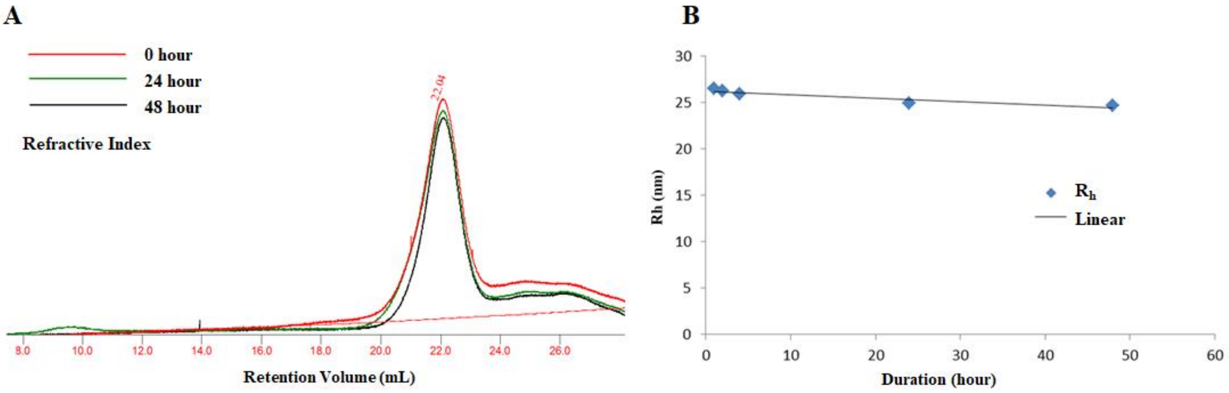

Figure S2. Stability of flaxseed kernel RG-I in 6 M NH_4OH: (A) HPSEC elution profile of KPI-ASF dissolving in 6 M NH_4OH after 0 h, 24 h & 48 h (concentration: 1 mg/mL), and (B) hydrodynamic radius (R_h) of KPI-ASF dissolving in 6 M NH_4OH after 1 h, 2 h, 4h, 24 h, & 48 h (concentration: 0.1 mg/mL)

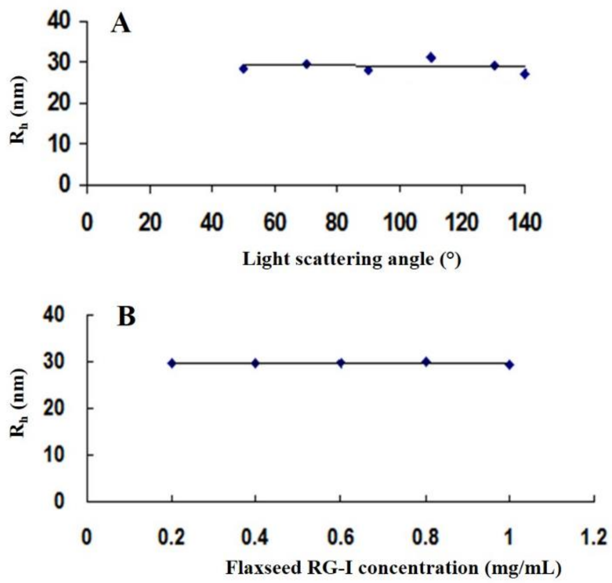

Figure S3. The mean hydrodynamic radius (R_h) of flaxseed mucilage RG-I: (A) the angular dependence (concentration: 0.2 mg/L) and (B) concentration dependence (at 90°) tests

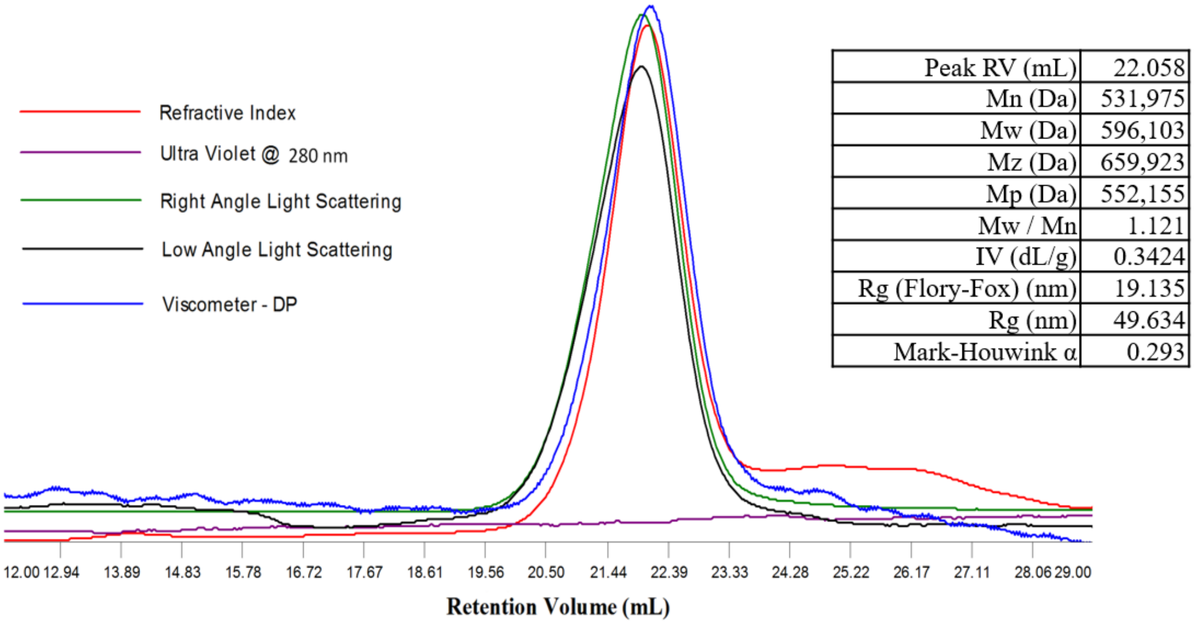

Figure S4. HPSEC elution profile of flaxseed kernel RG-I in 0.1 M $NaNO_3$ (concentration: 1 mg/mL, temperature: 40 °C)
